# Anatomical locations of air for rapid diagnosis of pneumothorax in blunt trauma patients

**DOI:** 10.1186/s13017-019-0263-0

**Published:** 2019-09-02

**Authors:** Ashraf F. Hefny, Fathima T. Kunhivalappil, Manoj Paul, Taleb M. Almansoori, Taoufik Zoubeidi, Fikri M. Abu-Zidan

**Affiliations:** 1 Trauma group, Department of Surgery, College of Medicine and Health Sciences, United Arab Emirates University, P O Box 18532, Al-Ain, United Arab Emirates; 2 Department of Radiology, Al Rahba Hospital, Abu Dhabi, United Arab Emirates; 3 Department of Radiology, College of Medicine and Health Sciences, United Arab Emirates University, Al-Ain, United Arab Emirates; 4 Department of Statistics, United Arab Emirates University, Al-Ain, United Arab Emirates

**Keywords:** Blunt, Injury, Pneumothorax, CT scan, Ultrasound

## Abstract

**Background:**

Bedside diagnostic ultrasound for traumatic pneumothorax is easy and reliable. However, the thoracic anatomical locations to be examined are debateable. We aimed to study the anatomical locations of blunt traumatic pneumothoraces as defined by chest CT scan to identify the areas that should be scanned while performing bedside diagnostic ultrasound.

**Methods:**

This is a retrospective analysis of a data collected for a previous study in blunt trauma patients at our hospital during a 4-year-period with CT confirmed pneumothoraces. The anatomical distribution of the pneumothoraces and their volume were analyzed. Advanced statistical analysis was performed using repeated measures logistic regression models.

**Results:**

Seven hundred three patients had a CT scan of the chest. Seventy-four patients (10.5%) were confirmed to have a pneumothorax. Only 64 were included in the study as they did not have a chest tube inserted before the CT scan. Twelve (18.8%) patients had bilateral pneumothorax. Seventy-six pneumothoraces were identified for which 41 patients had a right-sided pneumothorax and 35 patients had a left-sided pneumothorax. 95.1 % of the pneumothoraces detected on the right side were in the whole parasternal area with 75.6% seen in the lower parasternal region only. Similarly, 97.1 % of the pneumothoraces on the left side were seen in the whole parasternal area with 80% seen in the lower parasternal region only.

**Conclusions:**

The current study showed that air pockets of blunt traumatic pneumothoraces are mainly located at the parasternal regions especially in pneumothorax with small volume. We recommend a quick ultrasound scanning of the parasternal regions on both sides of the chest from proximal to distal as the appropriate technique for the detection of pneumothoraces in blunt trauma setting.

## Background

Early detection and treatment of pneumothorax in blunt trauma patients is crucial [1]. Simple pneumothorax can rapidly evolve to a life-threatening tension pneumothorax if not recognized at an early stage [2]. Conventional X-ray and computed tomography (CT) have been used to evaluate trauma patients with suspected pneumothorax.

Patients with multiple traumatic injuries are eventually brought to hospital in a supine position according to the guidelines of advanced trauma life support (ATLS) [3]. Accordingly, in this position, air accumulates anteriorly. This reduces the sensitivity of the supine chest X-ray in detecting underlying pneumothorax when compared to CT scan which is currently considered the gold standard tool in trauma settings [4, 5]. Nowadays, ultrasound (US) is a reliable tool in diagnosing traumatic pneumothorax and can be easily performed at the patients’ bedside. This is especially important in seriously injured patients who cannot be shifted to radiology departments for further cross-sectional imaging [6].

Focused assessment with sonography in trauma (FAST) has been effectively used to detect the presence of intraperitoneal fluid in trauma patients. Positioning of the ultrasound probe on the most dependable abdominal pouches helps in early and accurate detection of intraperitoneal fluid on FAST examination.

In traumatic pneumothorax, bedside usage of ultrasound proved to minimize the time taken for diagnosing pneumothoraces which led to early management and prevention of complications [1]. Therefore, the extended focused assessment with sonography in trauma (eFAST) has now been included in the ATLS guidelines. Yet, it remains unclear if a single or multiple locations should be scanned to provide an accurate diagnosis of pneumothorax in supine position [7].

The aim of this study is to determine the anatomical distribution of the intrapleural air and its commonest locations in blunt traumatic pneumothorax. The identification of these locations can guide the clinicians to develop an operating protocol of where to examine for the presence of pneumothorax when using ultrasound. This will help in timely improving the management and outcomes of traumatic pneumothorax.

## Methods

This study is a retrospective study with a post hoc analysis for a subgroup of patients from a previous study with CT confirmed pneumothoraces due to blunt trauma [8]. An in-depth analysis of the commonest anatomical air pockets distribution of pneumothorax was performed based on the results of the previously performed CT scans.

All patients who were presented to our hospital due to blunt traumatic injuries over a period of 4 years from October 2010 until October 2014 were studied. CT scans of the chest of all patients who had pneumothorax were reviewed. Patients who required a chest tube insertion before CT scan were excluded.

CT scans were performed using a General Electric 64 Slice Light Speed Volume (GE Health Care, USA). 2.5-mm thick axial images with intravenous contrast were obtained from the thoracic inlet down to the upper part of the abdomen. From the axial dataset, coronal and sagittal reformats were generated in the mediastinal, lung, and bone windows.

**Fig. 1:**
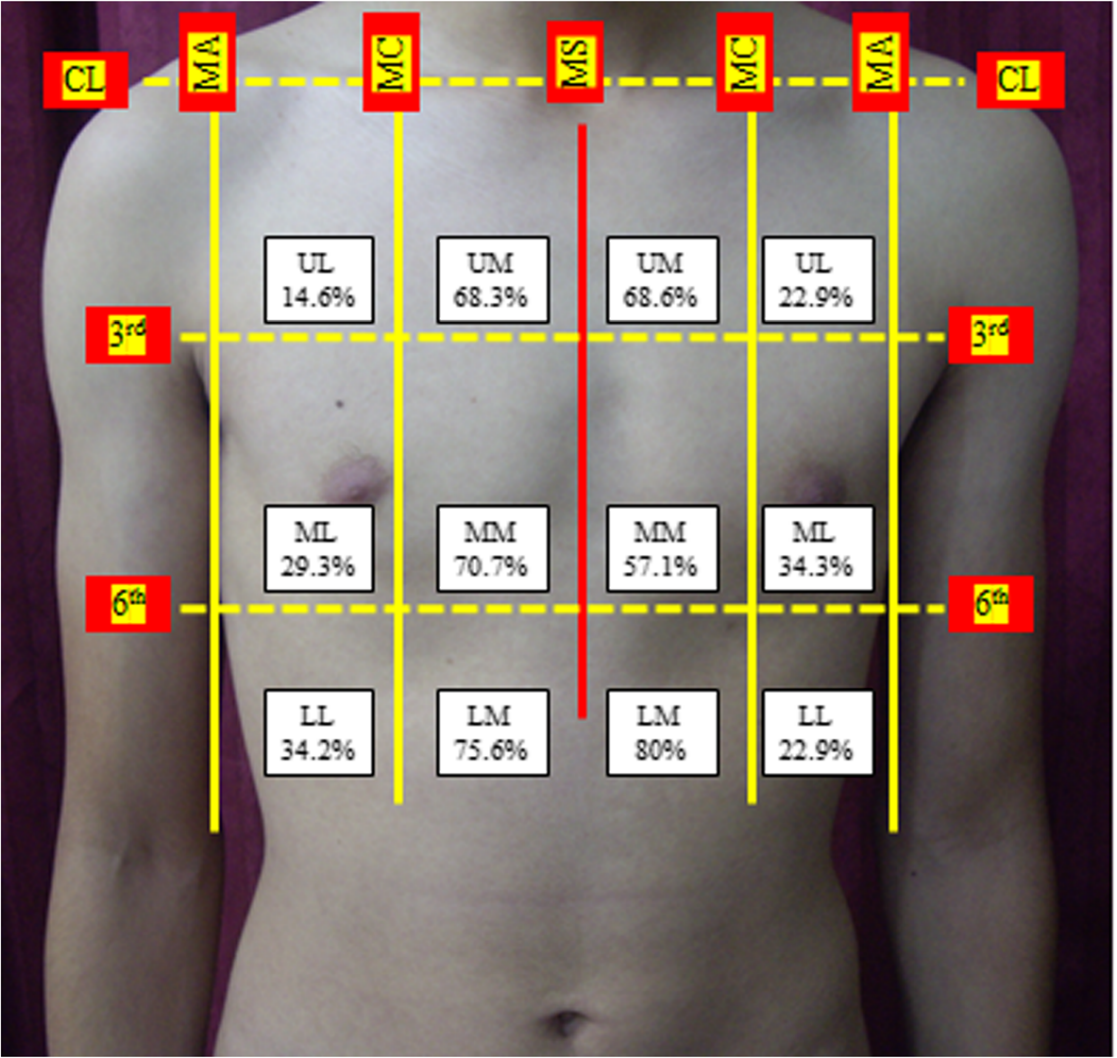
The percentage of air pockets in each hemithorax (right hemithorax 41 patients and left hemithorax 35 patients) according to a grid pattern dividing each hemithorax into six anatomical locations. CL, clavicular line; MC, midclavicular line; MA, midaxillary line; MS, midsternal line; 3rd, 3rd costosternal junction level; 6th, 6th costosternal junction level; UPS, upper parasternal; UL, upper lateral; MPS, middle parasternal; ML, middle lateral; LPS, lower parasternal; LL, lower lateral

Each hemithorax was divided into six anatomical regions including the anterior and lateral chest wall (Fig. 1). A grid pattern was formed by three horizontal lines at each hemithorax (the superior line at the clavicle level, middle line at the 3rd costosternal junction, and lower line at the 6th costosternal junction level), two vertical lines at each hemithorax (a medially positioned midclavicular line and a laterally positioned midaxillary line), and a vertical line at the middle of the sternum (midsternal).

This gird pattern divides each hemithorax into six areas namely upper parasternal (UP), upper lateral (UL), middle parasternal (MPS), middle lateral (ML), lower parasternal (LPS), and lower lateral (LL).

The formerly described grid pattern was developed to facilitate and precisely determine the volume and the location of air pockets in each hemithorax.

Volumetric analysis of an existing pneumothorax was calculated in milliliters (mL) using a preinstalled auto segmentation software (AW, GE Health Care, USA) which will accurately quantify its size in an objective manner [9, 10].

### Statistical analysis

Descriptive statistical analysis was performed. Each hemithorax was analyzed separately. Data on anatomical regions are presented by hemithorax, and the total percentage exceeds 100% because air pocket may involve more than one anatomical region.

The data was analyzed using the R statistical package. The probability of occurrence of a pneumothorax in a specific anatomical location among the six possible locations in the right or left lung was modeled using a repeated measures logistic regression model. The model was fitted and tested using the glmer function (lme4 package) in R.

The fixed effects of the logistic regression model included the standardized total volume of air in the pneumothorax locations and the locations of pneumothorax {1 = lower lateral (LL), 2 = lower parasternal (LPS), 3 = middle lateral (ML), 4 = middle parasternal (MPS), 5 = upper lateral (UL), 6 = upper parasternal (UPS)}.

Several covariance structures including unstructured and compound symmetry were compared for best fit in the model described above to account for correlation among the repeated measures. A covariance with compound symmetry was selected because it provided a better fit of the model to the data.

Al Rahba Hospital Research Ethics Committee has approved this research project (ARH/REC-040).

## Results

Chest CT scan was performed in 703 patients who presented to our institution because of blunt trauma. Seventy-four patients (10.5%) were identified to have blunt traumatic pneumothorax. Ten patients were excluded because they had a thoracostomy tube before CT scan performance and only 64 patients were included in the current study. Twelve (18.8%) patients had bilateral pneumothorax. Therefore, a total number of 76 pneumothoraces were studied including 41 (54%) right-sided pneumothoraces and 35 (46%) left-sided pneumothoraces. Thirty-three patients (51.6%) had a chest X-ray before CT scan, 28 patients (43.8%) had no evidence of pneumothorax (occult pneumothorax), and five patients (7.8%) had a pneumothorax.

There was no statistical difference in the volume of the pneumothorax between right and left hemithoraces (*P* = 0.64, Mann-Whitney *U* test). No patients had air in the posterior part of the pleural cavity.

### Right hemithorax

The CT scans of 41 patients with right-sided pneumothorax revealed air pockets within the three right parasternal regions (LPS, MPS, and UPS) in 39 patients (95.1 %) while 31 patients (75.6%) had air pockets at the lower parasternal region. Twenty patients (48.8%) had air pockets at the three lateral regions (LL, ML, and UL) (Fig. 1).

The significance of the fitted logistic regression model for the occurrence of a pneumothorax and its components, i.e., variables are shown in Table 1. which takes into account the six anatomical locations and the total volume of air in the pneumothorax. The probability of occurrence of pneumothorax depends significantly on the total volume of air, and it is more likely to occur in the parasternal locations (LPS, MPS, and UPS) than in the lateral ones (LL, ML, and UL) (*p* values < 0.001).

**Table 1:** Fixed effects of the repeated measures logistics regression of the occurrence of air pockets in blunt traumatic pneumothorax

Variable	Right lung		Left lung	
	Estimate	*P* value	Estimate	*P* value
Intercept	− 1.371	0.001	− 3.108	0.000
Volume	0.012	0.000	0.013	0.000
Location = LPS	2.046	0.000	4.024	0.000
Location = ML	− 0.291	0.591	1.181	0.143
Location = MPS	1.780	0.001	2.636	0.001
Location = UL	− 1.627	0.022	− 0.000007	0.999
Location = UPS	1.654	0.001	3.287	0.000
Location = LL	0^a^	–	0^a^	–

^a^Set to zero by design

*UPS* upper parasternal, *UL* upper lateral, *MPS* middle medial, *ML* middle lateral, *LPS* lower parasternal, *LL* lower lateral

**Fig. 2:**
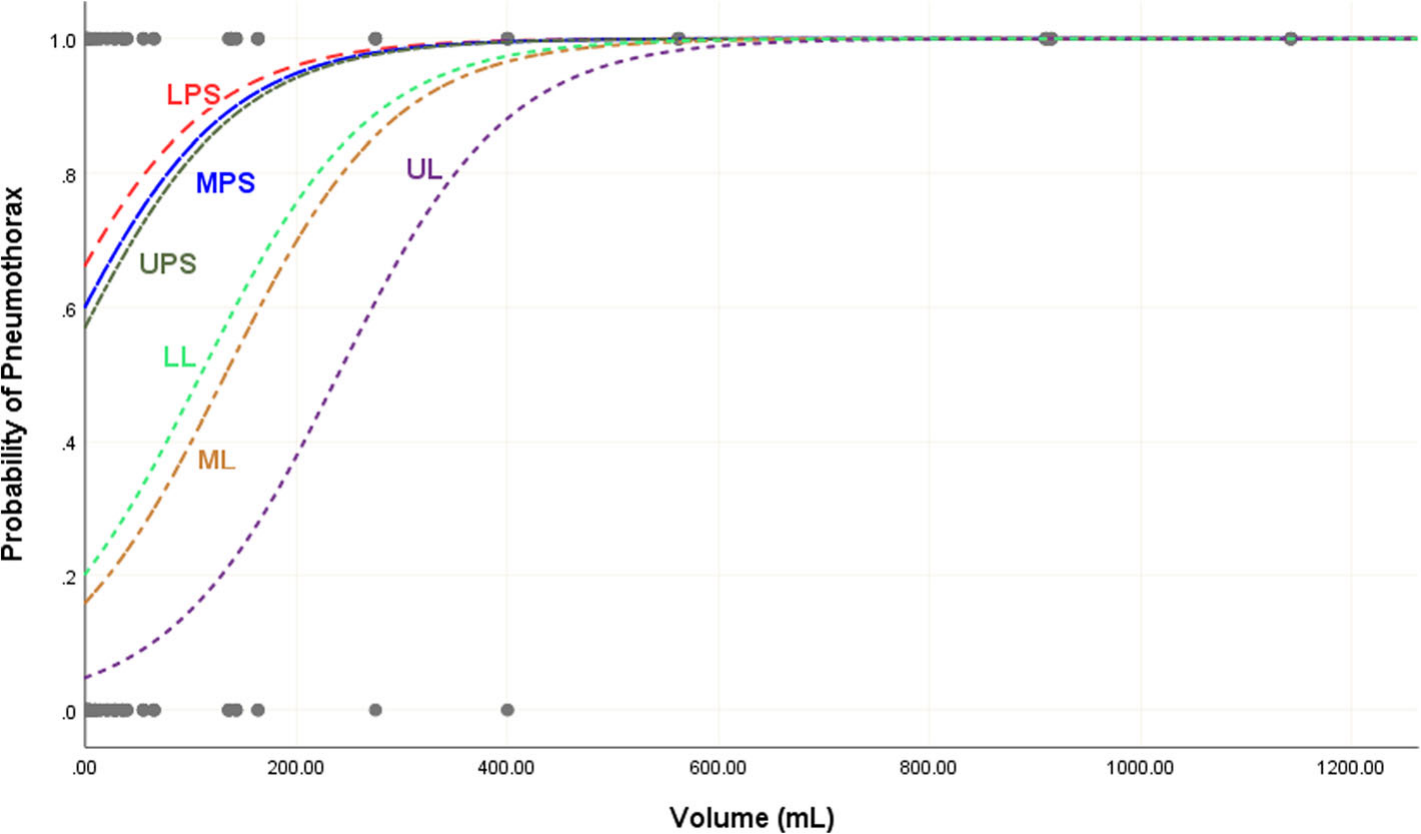
Estimated probability of occurrence of pneumothorax for each of the six location as a function of the total volume of air in the right hemithorax. UPS, upper parasternal; UL, upper lateral; MPS, middle parasternal; ML, middle lateral; LPS, lower parasternal; LL, lower lateral

The estimated probability of the occurrence of pneumothorax in each of the six locations as a function of the total volume of air is shown in Fig. 2. The data suggests that, irrespective of the total volume of air, pneumothorax is more likely to occur in any of the parasternal locations than in the lateral ones.

The probability of air pockets to be present in each location increases as the total volume of air increases. However, these probabilities are significantly higher in the parasternal locations than in the lateral ones especially when the total volume of air in the pneumothorax is less than 500 ml. That is, when the trauma results in a relatively large volume of air in the pneumothorax, it is equally likely to appear in all six anatomical locations, but when the total volume of air is less than 500 ml, then the parasternal locations are significantly more likely to receive the pneumothorax than the lateral ones.

### Left hemithorax

The CT scans of 35 patients with left-sided pneumothorax revealed air pockets within the three left parasternal regions (LPS, MPS, and UPS) in 34 patients (97.1 %) while 28 patients (80%) had air pockets at the lower parasternal region. Fourteen patients (40%) had air at the three lateral regions (LL, ML, and UL) (Fig. 1).

The fitted model for the occurrence of a pneumothorax in the left hemithorax and the estimates of the model coefficients are shown in Table 1. The probability of occurrence of pneumothorax depends significantly on the total volume of air, and it is more likely to occur in the parasternal locations (LPS, MPS, and UPS) than in the lateral ones (LL, ML, and UL), (*p* values < 0.001).

**Fig. 3:**
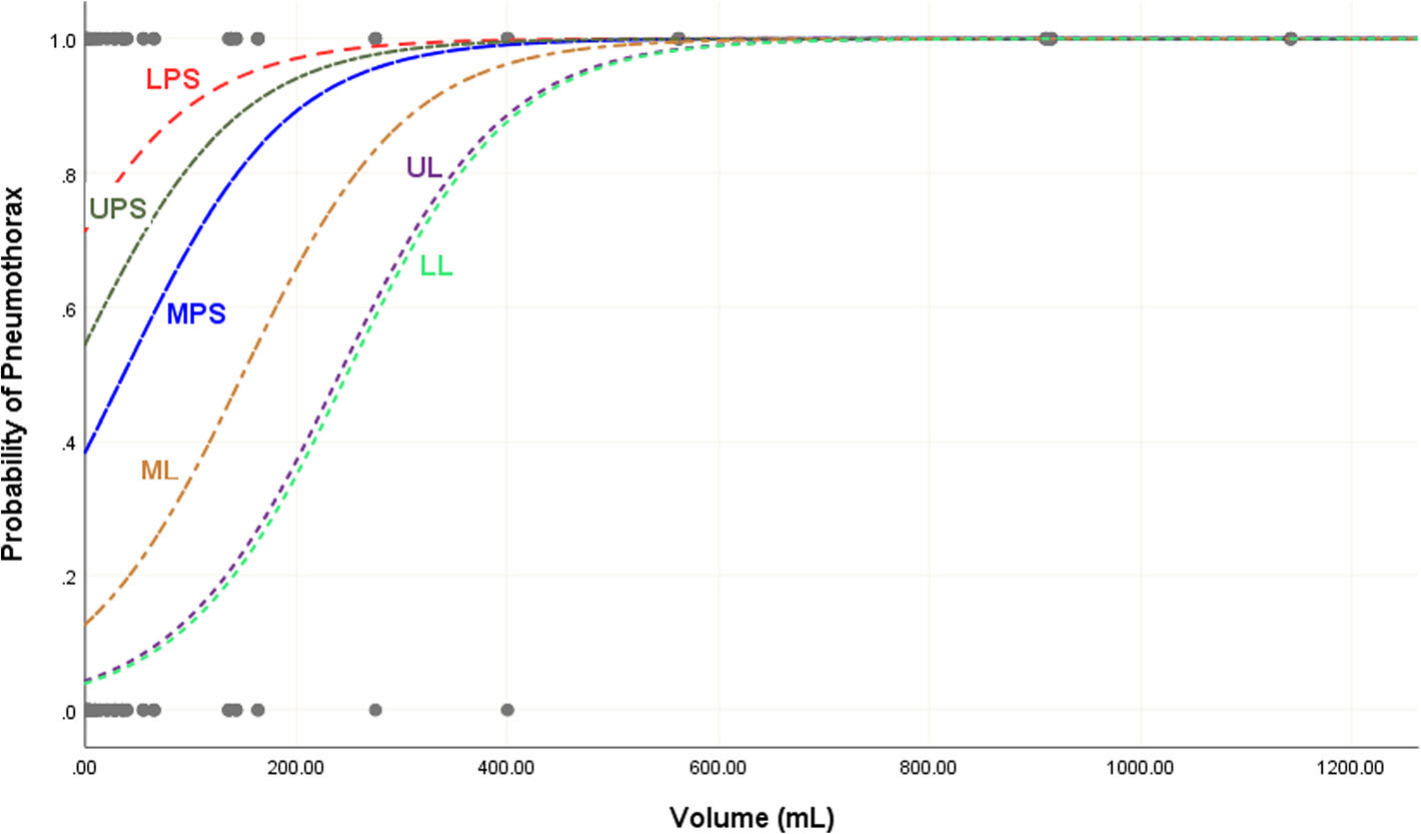
Estimated probability of occurrence of pneumothorax for each of the six location as a function of the total volume of air in the left hemithorax. UPS, upper parasternal; UL, upper lateral; MPS, middle parasternal; ML, middle lateral; LPS, lower parasternal; LL, lower lateral

The estimated probability of occurrence of air pockets in each of the six locations as a function of the total volume of air is shown in Fig. 3. The probability of air pockets to be present in each location increases as the total volume of air increases. However, these probabilities are significantly higher in the parasternal locations than in the lateral ones when the total volume of air in the pneumothorax is less than 500 ml. The lower and upper parasternal locations (LPS and UPS) had the highest probability of occurrence of air pockets.

## Discussion

The US of the chest has a comparable specificity to chest radiograph but is more sensitive in the detection of traumatic pneumothorax [5]. The current study showed that patients with blunt traumatic pneumothorax in supine position had a maximum air collection at the parasternal regions especially at the lower parasternal zones in both hemithoraces. At the time of examination of trauma patients, sonographic scanning of the parasternal regions will help in early and accurate detection of the existing pneumothorax.

To our knowledge, this study is the first in the literature to use the actual volume of the air in the blunt traumatic pneumothorax to determine the best locations for eFAST examination. Measuring the real size of air using automated or manual segmentation method could help in accurate localization of air pockets [11].

The peripheral and visceral pleura are fibrous tissue which appear on ultrasound as sliding white lines moving during respiration (lung sliding) [12, 13]. Presence of lung sliding will exclude a pneumothorax. Ultrasound indirectly identifies a pneumothorax when lung slide is not seen in the absence of other pathologies like lung collapse. The lung point, which is pathognomonic for a pneumothorax, is the point where sliding pleura meets a non-sliding pleura [14].

The sonographic features of pneumothorax will be more obvious where air maximally accumulates. In the literature, many controversies exist about the best locations for sonographic diagnosis of blunt traumatic pneumothorax. US scanning of every intercostal space between the clavicle and the diaphragm on each hemithorax were performed in the midclavicular line to detect pneumothorax [15]. This method is time-consuming and less practical in trauma setting as it wastes a critical time needed for the management of other serious injuries. Other studies, based on the assumption that air will accumulate anteriorly in supine patients, have recommended US scanning the chest in one or two positions: either anterior at the second intercostal space in the midclavicular line [16, 17] and/or at the anterolateral chest wall at the 4th or 5th intercostal space at midaxillary line [5, 18].

Similar to another study, our study has shown that maximum air collections were at the parasternal regions [1]. Scanning the parasternal region by quick sweeping can detect 95% of pneumothorax on right hemithorax and 97% on the left side. Detection of pneumothoraces increases by moving from lateral to medial sites (towards the parasternal regions) [6].

Development of pneumothorax is a dynamic process for which small pneumothoraces can progress and increase in size over time, leading to respiratory distress. Our study has clearly shown that air collections of smaller size (less than 500 mL) are better detected on the parasternal region (Figs. 2 and 3) which will help in early detection of small pneumothoraces. In contrast with other studies [5, 18], our study has shown that the lateral sites had the least probabilities for the presence of air pockets. Moreover, the presence of air at these sites indicates large pneumothorax volume (more than 500 mL).

On the right-sided pneumothorax, there was no statistical difference in the presence of air pockets at the three parasternal regions while on the left side, the lower and upper parasternal locations had the highest probability. This can be explained by the effect of the anatomical position of the heart and mediastinum on the left middle parasternal region.

### Limitations

This is a retrospective single-center study with a relatively small sample size. It only included patients with pneumothorax who had no chest tube placement before radiological imaging with CT scan. Unstable trauma patients would not have been captured before chest tube insertion. Therefore, we cannot generalize these findings to all blunt traumatic pneumothorax patients. Yet, patients who had a chest tube placement before CT scanning might have larger pneumothorax which was detected clinically or by chest radiography.

The results of eFAST in trauma patients depend on the experience of the operator, quality of the ultrasound machine, patient’s body habitus, and the presence of surgical emphysema [16]. Our findings regarding the anatomical distribution of intrapleural air in blunt traumatic pneumothorax are based on the analysis of CT scan images only because eFAST was not performed [19]. So, a prospective study correlating the CT scan findings of this study with an actual eFAST is needed. Such a study will have a high impact on clinical practice by determining the benefits of sonographic scanning based on the result of the current study.

## Conclusions

The current study showed that air pockets of blunt traumatic pneumothoraces are mainly located at the parasternal regions especially in pneumothorax with small volume. We recommend a quick ultrasound scanning of the parasternal regions on both sides of the chest from proximal to distal as the appropriate technique for the detection of pneumothoraces in blunt trauma setting.

## Data Availability

There is no additional data available to share with the readers. Data can be shared with the Editor of the Journal if requested.

## Abbreviations

3rd: Third costosternal junction level
6th: Sixth costosternal junction level
ATLS: Advanced trauma life support
CL: Clavicular line
CT: Computed tomography
eFAST: Extended focused assessment with sonography in trauma
LL: Lower lateral
LPS: Lower Parasternal
MA: Midaxillary line
MC: Midclavicular line
ML: Middle lateral
MPS: Middle parasternal
MS: Midsternal line
UL: Upper lateral
UPS: Upper parasternal
US: Ultrasound
